# Rhinovirus wheezing illness in infancy is associated with medically attended third year wheezing in low risk infants: results of a healthy birth cohort study

**DOI:** 10.1002/iid3.77

**Published:** 2015-08-27

**Authors:** Janneke J. H. de Winter, Louis Bont, Berry Wilbrink, Cornelis K. van der Ent, Henriette A. Smit, Michiel L. Houben

**Affiliations:** ^1^ Department of Pediatrics, University Medical Center UtrechtUtrechtThe Netherlands; ^2^ Department of Clinical Immunology and Rheumatology, Academic Medical CenterUniversity of AmsterdamAmsterdamThe Netherlands; ^3^ Laboratory of Infectious Diseases and Perinatal Screening, National Institute of Public Health and the EnvironmentBilthovenThe Netherlands; ^4^ Julius Center for Health Sciences and Primary Care, University Medical Center UtrechtUtrechtThe Netherlands

**Keywords:** Rhinovirus, wheezing, asthma, infant, respiratory virus

## Abstract

Rhinoviruses may be pathogens contributing to the development of childhood wheezing. However, their role in low risk infants without an asthmatic predisposition is unknown. Knowing which healthy, low risk children are at increased risk for childhood wheezing after rhinovirus wheezing illness (RV‐WI) in infancy, might help in developing prevention and treatment strategies for childhood wheezing. The aim of this study was to determine the association of medically attended wheezing at the age of three with RV‐WI in the first year of life in low risk children without parental asthma. In a low risk, prospective birth cohort study, we followed 181 healthy born children from birth through the third year of life. We considered children ‘low risk’ if neither parent had a doctor's diagnosis of asthma. We determined infant RV‐WI by parent‐reported wheezing (based on daily logs) and simultaneous molecular rhinovirus detection in the first year of life. Respiratory function and blood eosinophil count were both measured in the first month of life. The primary outcome, third year wheezing, was defined as the use of prescribed inhaled asthma medications together with a doctor's visit for respiratory symptoms in the third year of life. We calculated the association of RV‐WI with medically attended third year wheezing and other known possible risk factors for wheezing at the age of three. Among low risk children, third year wheezing was observed in 7 out of 18 (39%) children with versus 10 out of 163 (6%) children without infant RV‐WI (OR 9.7, 95% CI 3.1–33.5, *P *< 0.0001). The association between RV‐WI and third year wheezing was unchanged after adjustment for potential confounders such as eosinophilia and atopic eczema. RV‐WI is a robust and independent risk factor for third year wheezing in low risk children without parental asthma. Future research will identify and protect those children at increased risk for RV‐WI.

## Introduction

Rhinoviruses are increasingly recognised as the most common pathogens causing lower respiratory tract infections 1, 2 and acute wheezing in infancy 3, 4, 5, 6, 7, 8, 9, 10, 11, 12. In a community‐based cohort study, we previously found that rhinoviruses accounted for 42% of all lower respiratory tract infections 13. Furthermore, rhinoviruses may also play a role in the development of subsequent wheezing and asthma 14, 15, 16, 17, 18. However, the role of rhinoviruses in wheezing and asthma after rhinovirus wheezing illness (RV‐WI) is not clear. Are rhinoviruses a marker of children predisposed to wheezing and asthma or do rhinoviruses interfere with normal lung development or immune maturation? Understanding the link between rhinoviruses and asthma might enable the development of early prevention and treatment strategies for wheezing and asthma.

Previous research has shown that RV‐WI is a strong predictor of subsequent wheezing and asthma, especially in high risk children with an atopic background 19, 20, 21 and in children hospitalized for wheezing 14, 18, 22, increasing the risk of asthma even at adolescent age 23. However, little is known about the short‐ and long‐term morbidity after RV‐WI in healthy infants lacking an *a priori* increased risk of asthma. If healthy infants are at increased risk of developing wheezing after RV‐WI in infancy, we could identify, follow‐up and possibly treat those at high risk for RV‐WI. Therefore the aim of this study was to answer the following questions: (1) what is the association of wheezing at the age of three after infant RV‐WI in children without parental asthma? (2) Is this association influenced by known risk factors for wheezing such as obstructive respiratory function at one month of age, atopic characteristics or known environmental risk factors for wheezing? To answer these questions, we followed healthy children without parental asthma from birth until the age of three in a prospective, community‐based birth cohort study.

## Methods

### Study design and population

In a prospective, community‐based birth cohort study, children born in the University Medical Center Utrecht and the Diakonessen Hospital Utrecht were included. Study design and recruitment criteria have been published previously 24. In short, healthy children, without any birth defects or illnesses at birth, born after an uncomplicated pregnancy after 37 weeks of gestation were enrolled at birth and followed prospectively. The institutional review boards of the University Medical Center Utrecht and the Diakonessen Hospital Utrecht approved the study protocol. All parents gave their written informed consent.

### Subject characteristics

Demographic, obstetric, atopic and environmental characteristics were assessed using data from the hospital delivery files (gender, gestational age, month of birth and birth weight) and from standardized questionnaires completed by parents at one month, one year and three years of age as described in more detail previously 24. Furthermore, general practitioners completed questionnaires on respiratory and atopic diagnoses both in the first and third year of life. During the first year of life, parents recorded data on respiratory symptoms in a log on a daily basis, including symptoms of wheezing, as described previously 13. All parents received live oral and written instructions on how to carefully record respiratory symptoms and how to discern wheezing from other breathing sounds. Parents were trained by a physician to collect a nasopharyngeal swab at the second day of all first year episodes of respiratory symptoms. All log data were reviewed for the presence of episodes of respiratory tract infection by two researchers, blinded for the results of virologic tests 13.

### Clinical measurements

At one month of age (median age 34 days, IQR 30–39 days), respiratory function was tested and blood was drawn to measure the absolute eosinophil count. The single occlusion technique was used to measure compliance and resistance 25, 26, 27. An experienced nurse assessed the respiratory function test during natural sleep and without any sedatives. The results for compliance (Crs) and resistance (Rrs) of the respiratory system were transformed logarithmically and standardized by correction for length, weight and age, yielding Z‐scores (normally distributed). Generally, a high compliance and a low resistance are considered beneficial.

Nasopharyngeal swab samples were sent in 2 mL of viral transport medium and frozen at −80°C until polymerase chain reaction (PCR) assays were performed. Trained analysts performed real time PCR assays to detect viral pathogens such as rhinoviruses and respiratory syncytial virus (RSV) as described in more detail previously 24.

### Definitions

Parental asthma was defined as asthma ever diagnosed by a physician in one or both parents. RV‐WI in the first year of life was defined as the simultaneous presence of parent‐reported wheezing for ≥ 2 days (based on daily logs) and molecular rhinovirus detection in viral samples collected during respiratory episodes in the first year of life 24. Rhinovirus respiratory tract infection (RV‐RI) was defined as parent‐reported mild to severe cough for ≥ 2 days and molecular RV detection irrespective of the presence of wheezing in the first year of life. RSV lower respiratory tract infection (RSV LRTI) was defined as the simultaneous presence of parent‐reported wheezing or moderate to severe cough for ≥ 2 days and molecular RSV detection in the first year of life.

The primary outcome of the study, medically attended third year wheezing, was defined as the use of prescribed inhaled asthma medications (corticosteroids and/or β_2_‐sympathomimetics) together with a doctor's visit for respiratory symptoms in the third year of life. Respiratory symptoms were defined as any kind of respiratory symptoms for which parents felt the urgency to visit the general practitioner. The secondary outcome, physician‐diagnosed wheezing, was composed of a doctor's diagnosis of wheezing or the use of prescribed inhaled asthma medications (corticosteroids and/or β_2_‐sympathomimetics) in the third year of life. A doctor's diagnosis of wheezing was defined according to the International Classification of Primary Care (ICPC) of either wheezing (R03) or asthma (R96).

### Confounding variables

Potential confounding variables were defined *a priori* based on known and hypothesized risk factors for wheezing. Factors included were birth weight, gestational age 28, male sex 29, 30, having ≥1 siblings 29, maternal smoking during pregnancy 29, 31, the presence of ≥ 1 highly educated parent, day care attendance 29, being breastfed ever, respiratory function at one month of age 29, 32, 33, atopic eczema in infancy 34, 35, blood eosinophil count at one month of age 34, 35, RSV LRTI during infancy 21 and the presence of parental atopic eczema or allergic rhinitis 30. We defined high education as having at least a bachelor's degree. Day care attendance was defined as any day care attendance during the first year of life. Atopic eczema was defined as a physician's diagnosis of atopic eczema during the first year of life.

### Statistical analyses and handling of missing values

We calculated the ratio of the odds of wheezing in the third year of life for children with RV‐WI in the first year of life over those without RV‐WI in the first year. Generalized linear models were used to estimate the 95% confidence interval (CI) for this odds ratio and to adjust for confounders. We included the two variables showing the strongest association with RV‐WI in regression models as potential confounders. Results are presented as odds ratio with the associated 95% confidence interval (CI) and *P*‐value. Results were considered significant if the 95% CI did not include 1 and the *P*‐value was <0.05.

Since missing values usually do not occur randomly, exclusion of participants with missing values (complete case analysis) may result in biased estimates. Therefore we used single imputation techniques (regression analysis) to address missing values, including missing outcome values. Respiratory function was measured successfully in 81 of 181 children (45%) and respiratory function measurements were therefore not imputed. The major reasons for failure of respiratory function measurement were technical issues (insufficient quality and / or number of occlusions) (42%) and no sleep or a too short period of sleep (52%). Other missing values did not exceed 5%, except for day care attendance (9%), atopic eczema (19%) and blood eosinophil count (20%).

## Results

We found that RV‐WI during the first year of life was highly associated with third year wheezing, also after adjustment for known risk factors for wheezing. The main characteristics of the children with and without RV‐WI in the first year of life are summarized in Table 1.

**Table 1:** Characteristics of the third‐year study population according to infant rhinovirus wheezing illness status

	Rhinovirus wheezing illness in the first year of life		
	No (*n *= 163)	Yes (*n *= 18)	*P*‐value
Birth weight (kg)	3.6 (0.5)	3.7 (0.5)	0.61
Gestational age (weeks)	40.0 (1.0)	40.3 (0.9)	0.42
Male	85 (52)	8 (44)	0.54
Siblings	99 (61)	11 (61)	0.98
Maternal smoking during pregnancy	8 (5)	1 (6)	0.91
Highly educated parent(s)	134 (82)	12 (67)	0.11
Day care attendance	108 (66)	9 (50)	0.17
Breastfeeding	151 (93)	15 (83)	0.17
RSV LRTI	17 (10)	1 (6)	0.51
Ln Compliance	0.05 (1.06)	−0.45 (1.12)	0.11
Ln Resistance	0.07 (0.98)	−0.20 (1.24)	0.37
Atopic characteristics			
Atopic eczema during infancy	44 (27)	10 (56)	0.01
Blood eosinophil count (For the eosinophile count 10^9^/L)	0.45 (0.39)	0.80 (0.55)	0.02
Hay fever mother	23 (14)	2 (11)	0.71
Hay fever father	27 (17)	3 (18)	0.94
Eczema mother	29 (18)	5 (28)	0.32
Eczema father	15 (9)	0 (0)	0.19

Values represent mean (SD) or frequency (%). Respiratory function and blood eosinophil count were both measured at one month of age. RSV LRTI: respiratory syncytial virus lower respiratory tract illness, Ln Compliance and Resistance: compliance and resistance of the respiratory system, expressed on a natural logarithmic scale and standardized for length, weight and age (Z‐score).

### Study population and infant rhinovirus wheezing illness

**Figure 1:**
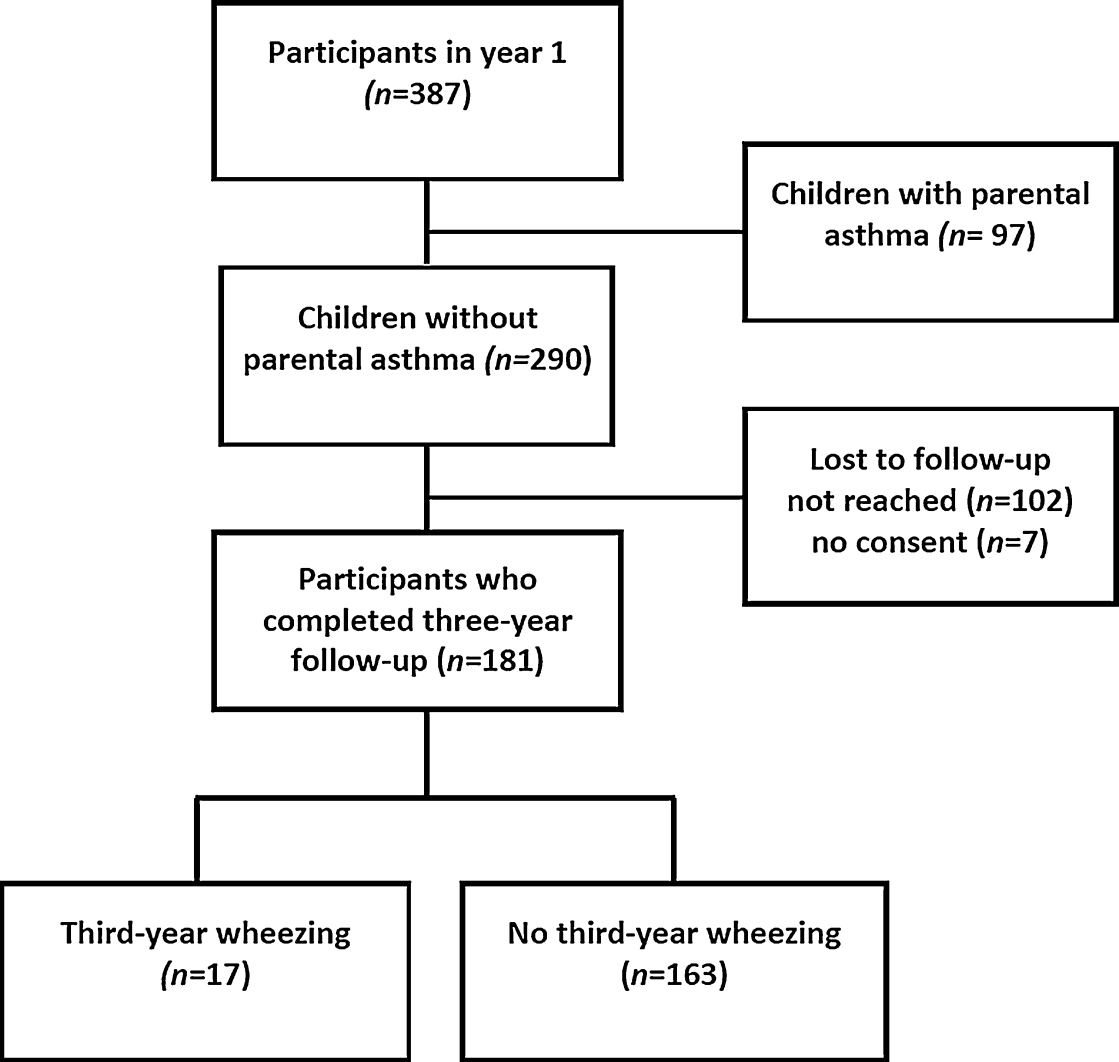
Flow chart of children included in the prospective birth cohort study.

We followed 387 neonates throughout the first year of life, including 290 children without parental asthma. Of those 290 children, 181 (62%) completed three‐year follow‐up (Fig. 1). The remaining 181 children were representative for the whole study group (Supplementary Table 1). Of those 181 children, 51% were male. The mean birth weight was 3.6 kg (SD 0.5) and the mean gestational age was 40 weeks (SD 1.1). Of the 97 children with parental asthma, 49 (51%) completed three‐year follow‐up.

The 181 children who completed three‐year follow‐up reported 271 episodes of respiratory tract infections in the first year of life. Eighteen out of 181 children (10%) developed RV‐WI in the first year of life and 65 out of 181 children (36%) developed RV‐RI. No significant differences were observed between infants who did and who did not develop RV‐WI in the first year of life regarding known possible confounders: male sex, gestational age, respiratory function, number of siblings, RSV LRTI, day care attendance, maternal smoking, high level of education of one or both parents and breastfeeding. The RV‐WI group however had a higher mean eosinophil count (0.80 * 10^9^/L (SD 0.39 * 10^9^/L) vs. 0.45 * 10^9^/L (SD 0.55 * 10^9^/L), *P *= 0.02), measured at one month of age and a higher prevalence of atopic eczema during the first year of life than the group without RV‐WI (56% vs. 27%, *P = *0.012) (Table 1).

### Third year wheezing

**Figure 2:**
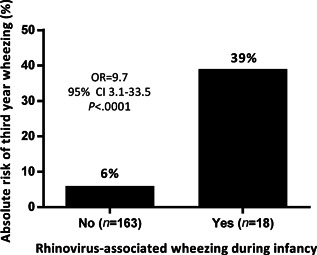
Association between medically attended wheezing in the third year of life after rhinovirus wheezing illness in infancy in 181 low risk children. Association between medically attended wheezing in the third year of life in groups with and without rhinovirus wheezing during infancy. OR: unadjusted odds ratio. 95% CI 95%: confidence interval.

RV‐WI in the first year of life was highly associated with wheezing in the third year of life: of the 18 children with RV‐WI in the first year of life, 7 (39%) developed wheezing during the third year of life, compared to 10 out of 163 (6%) children who did not have RV‐WI in the first year of life (unadjusted OR 9.7, 95% CI 3.1–33.5, *P *< 0.0001). Children with and without third year wheezing did not differ in gestational age, sex, number of siblings, daycare, breastfeeding, parental atopic characteristics, atopic eczema in the third year of life, blood eosinophil count at one month of age and respiratory function at one month of age (data not shown). The children with wheezing during the third year of life did have a higher percentage of atopic eczema during infancy (11 (65%) vs. 55 (27%), *P *= 0.001) (Fig. 2).

Third year wheezing was not associated with RV‐RI (OR 0.6, 95% CI 0.2–2.1, *P *= 0.38). One out of 17 children with third year wheezing (6%) had RSV LRTI during infancy, compared to 17 out of 163 children without third year wheezing (10%, OR 0.5, 95% CI 0.1–4.3).

When we adjusted the third‐year wheezing risk after RV‐WI for eczema in infancy and for blood eosinophil count at one month of age, the risk was not influenced (adjusted OR 13.6, 95% CI 2.2–83.1, *P *= 0.005).

A sensitivity analysis using the alternative outcome physician‐diagnosed wheezing showed similar results. Third‐year physician‐diagnosed wheezing was diagnosed in 7 out of 18 (39%) of children with RV‐WI in the first year of life and in 22 out of 163 (13%) children without RV‐WI in the first year of life (OR 4.1, 95% CI 1.4–11.6, *P *= 0.005).

## Discussion

The results of our study suggest that, in low risk children without a background of parental asthma, rhinovirus wheezing illness (RV‐WI) during infancy is strongly and independently associated with wheezing at the age of three. The increased risk seems to be independent from known risk factors for wheezing, such as early infant eosinophilia, obstructive respiratory function, or atopic eczema in the first year of life.

Our findings in low risk children are consistent with results of previous studies, showing the role of rhinoviruses as contributors in wheezing and asthma later in life in high‐risk children or in children hospitalized for wheezing. The Childhood Origins of ASThma cohort (COAST), a high‐risk cohort of 285 children with at least one parent with respiratory allergies, demonstrated that RV‐WI during infancy was the strongest predictor of wheezing at 3 years of age (OR 6.6). Furthermore, RV‐WI during infancy increased the asthma risk at 6 years of age independently from aeroallergen sensitization 19, 20. Another prospective high risk study by Kusel *et al.* showed similar results; RV‐WI was associated with persistent wheezing and asthma (OR 2.5 and 2.9, respectively) 21. In a Finnish cohort of 82 prospectively followed infants who were hospitalized for wheezing, Kotaniemi‐Syrjänen and colleagues showed that RV‐WI was associated with increased bronchial responsiveness at school age (OR 6.5) 22, 36. The VINKU‐study, another Finnish cohort of 263 hospitalized children for bronchiolitis, showed that sensitization is linked to RV‐WI, but not to acute wheezing induced by other viruses 37. Jartti and coworkers recently showed that children admitted with RV bronchiolitis more often had a history of wheezing than children admitted with bronchiolitis of other viral etiology 12. In another recent study, Midulla *et al.* showed that recurrent wheezing after hospitalization for bronchiolitis was associated with RV and not with other viral etiology 18. Although the risk of third year wheezing after RV‐WI in our cohort study is comparable to that risk in other studies, the prevalence of third year wheezing is lower in our cohort study (17 out of 181 children, 9.4%). The relatively low prevalence of third‐year wheezing might be due to the fact that we chose a ‘low risk’ cohort: we followed children of parents without asthma, who were not hospitalized for infant wheezing illnesses. Similarly, the difference in the number of infant RV‐WI amongst children with third year wheezing between our cohort study and the COAST 19 (41 vs. 63%, respectively) might also be explained by this difference in study population.

The RV‐WI group had a higher eosinophil count at one month of age, and a higher prevalence of atopic eczema during the first year of life than the group without RV‐WI. Eosinophilia was independently associated with and temporarily followed by RV‐WI, since RV‐WI did not occur before the age of one month. This suggests that eosinophilia (and probably infant eczema) and RV‐WI have a synergistic effect on wheezing later in childhood. However, since correcting for eosinophila and infant eczema did not decrease the effect of RV‐WI on wheezing in the third year of life, eosinophilia and infant eczema alone cannot fully explain the risk of wheezing in the third year of life. A population‐based cohort study empowered to study the interaction between atopy, RV‐WI and wheezing later in childhood could answer the question if this synergistic effect exists.

Respiratory function measured at one month of age did not significantly differ between infants with and without RV‐WI in infancy. However, a trend towards lower compliance of the respiratory system in infants with RV‐WI in infancy was found. The insignificance might be due to small sample size. A recent unselected birth cohort study of Van der Gugten *et al.* showed similar results: children with a decreased respiratory function early in life were at increased risk of developing both RV‐WI as well as childhood wheezing 33.

The strengths of this study are 1 the three‐year prospective follow‐up from birth until the age of three, 2 the detailed data on demographic, virological, environmental and atopic characteristics collected at several time points by both parents and physicians and 3 the respiratory function test and blood eosinophil count after one month of life before any airway symptoms arose. Thereby, we were able to correct for the impact of early possible risk factors of wheezing.

The limitations of this study should be discussed. First, we only performed PCR assays during respiratory episodes. Hereby we can only speculate about the causal relationship between the presence of RV and wheezing illness. However, this relation appeared to be specific, and was absent for rhinovirus respiratory tract infection without wheezing (RV‐RI). Accordingly, in a recent unselected birth cohort study, Van der Gugten *et al.* showed that RV presence itself was not associated with wheezing at age 4, while RV‐WI was 33. A recent study showed that IL‐33 and type 2 cytokines are induced during RV‐induced asthma exacerbations 38, suggesting that RV plays a key role in amplifying type 2 inflammation. Another experimental RV‐induced asthma exacerbation model showed an increase in neutrophil‐attracting CXCL8 and IL‐8 39. Both IL‐33 and CXCL8/IL‐8 are promising new targets for prevention and therapy of wheezing and asthma.

Second, only 181 of 290 children (61%) completed the follow‐up. However, baseline characteristics of participants and of children that were lost‐to‐follow‐up were similar, thereby reducing the risk of selection bias (see Supplementary table 1).

Third, to create a population of children without an *a* *priori* increased asthma risk, we followed a low risk birth cohort, which we defined as non‐parental asthma. Although several studies show increased risks of atopy and asthma due to genetic factors 40, 41, these children are not per definition at low risk of developing asthma.

Fourth, viral swabs were in this study performed by parents. This might be considered a limitation of this study. However, in a randomized study Van der Zalm *et al*. showed that parental viral swab collection results in higher sampling yields when compared to swab collection by a trained nurse, without any difference in severity of symptoms or loss of viral recovery rate 42.

After prolonged follow‐up of these low risk children, establishment of rhinovirus as an independent risk factor of childhood wheeze and / or asthma would generate several ways to predict and prevent early childhood respiratory infections and wheeze / asthma. Careful follow‐up of the individuals at risk and, possibly, preventive treatment will hopefully be available in the near future.

## Conclusion

We conclude that RV‐WI in infancy is a robust risk factor for wheezing at the age of three in children without parental asthma, independent from other known risk factors for wheezing. Further follow‐up will show whether these children will have an increased asthma risk at school age. Ultimately, preventive treatment, developed to modify immune responses to viral infection or to prevent rhinovirus infection itself may help.

## Conflict of Interest

LB received research funds or consultancy fees from AbbVie, MedImmune, Gilead, Ablynx JanssenPharmaceuticals (all received by institution).

## Supporting information

Additional supporting information may be found in the online version of this article at the publisher's web‐site.

**Table S1**. Characteristics of the Study Population.
