# Supplementary material for: Rhinovirus wheezing illness in infancy is associated with medically attended third year wheezing in low risk infants: results of a healthy birth cohort study
Source: Immun Inflamm Dis. 2015 Aug 27;3(4):398–405. doi: 10.1002/iid3.77 (PMC4693725; doi:10.1002/iid3.77)
Supplement: Supplementary file 1 — Table S1. Characteristics of the Study Population. [file IID3-3-398-s001.doc]

**Supplementary table 1. Characteristics of the Study Population**

| **Characteristic** |  | | |
| --- | --- | --- | --- |
|  | **Participants in year 1**  ***n=*387** | **Children without parental asthma *n=*290** | **Children who completed three-year follow-up**  ***n*=181** |
| Birth weight (kg) | 3.6 (0.5) | 3.6 (0.5) | 3.6 (0.5) |
| Gestational age (weeks) | 40.0 (1.0) | 40.0 (1.1) | 40 (1.1) |
| Male | 196 (51) | 150 (51) | 93 (51) |
| Siblings | 235 (61) | 181 (62) | 110 (61) |
| Maternal smoking during pregnancy | 37 (10) | 29 (10) | 9 (5) |
| Highly educated parent(s) | 268 (73) | 207 (73) | 137 (80) |
| Day care attendance | 181 (62) | 145 (63) | 102 (65) |
| Breastfeeding | 231 (86) | 183 (87) | 135 (92) |
| RSV LRTI during infancy | 30 (8) | 37 (13) | 18 (10) |
| RV-WI during infancy | 27 (12) | 29 (10) | 18 (10) |
|  |  |  |  |
| ***Atopic characteristics*** |  |  |  |
| Atopic eczema during infancy | 81 (30) | 57 (27) | 43 (30) |
| Eosinophil count (*10^9^/L) | 0.47 (0.38) | 0.48 (0.39) | 0.47 (0.41) |
| Allergic rhinitis mother | 76 (20) | 48 (17) | 25 (14) |
| Allergic rhinitis father | 71 (19) | 49 (17) | 30 (17) |
| Atopic eczema mother | 72 (19) | 50 (17) | 34 (19) |
| Atopic eczema father | 45 (12) | 24 (8) | 15 (9) |
|  |  |  |  |

**Values represent mean (SD) or frequency (%). No statistical differences between subgroups were detected (*P*=0.05). RSV LRTI: respiratory syncytial virus lower respiratory tract illness. RV-WI: rhinovirus wheezing illness**
